# Trajectories of Dietary Patterns and Their Associations with Overweight/Obesity among Chinese Adults: China Health and Nutrition Survey 1991–2018

**DOI:** 10.3390/nu13082835

**Published:** 2021-08-18

**Authors:** Jiguo Zhang, Huijun Wang, Zhihong Wang, Feifei Huang, Xiaofan Zhang, Wenwen Du, Chang Su, Yifei Ouyang, Li Li, Jing Bai, Bing Zhang, Shufa Du, Gangqiang Ding

**Affiliations:** 1 Department of Public Nutrition, National Institute for Nutrition and Health, Chinese Center for Disease Control and Prevention, Beijing 100050, China; zhangjg@ninh.chinacdc.cn (J.Z.); wanghj@ninh.chinacdc.cn (H.W.); wangzh@ninh.chinacdc.cn (Z.W.); huangff@ninh.chinacdc.cn (F.H.); zhangxf@ninh.chinacdc.cn (X.Z.); duww@ninh.chinacdc.cn (W.D.); suchang@ninh.chinacdc.cn (C.S.); ouyyf@ninh.chinacdc.cn (Y.O.); lili@ninh.chinacdc.cn (L.L.); baijing@ninh.chinacdc.cn (J.B.); zhangbing@chinacdc.cn (B.Z.); 2 Department of Nutrition and Carolina Population Center, University of North Carolina at Chapel Hill, Chapel Hill, NC 27599, USA; dushufa@unc.edu

**Keywords:** dietary patterns, trajectories, overweight, obesity, Chinese adults

## Abstract

It is essential to understand the impact of different dietary pattern trajectories on health over time. Therefore, we aimed to explore the long-term trajectories of dietary patterns among Chinese adults and examine the prospective association between different trajectory groups and the risk of overweight/obesity. The sample was 9299 adults aged 18 years or older from the China Health and Nutrition Survey (CHNS) between 1991 and 2018. We used factor analysis to identify dietary patterns and group-based trajectory modeling to identify dietary pattern trajectories. Three trajectories of a southern pattern and a modern pattern and four trajectories of a meat pattern were identified. Participants who followed the highest initial score and a slight decrease trajectory (OR = 1.63; 95% CI: 1.04, 2.54) of the meat dietary pattern were positively associated with risk of overweight/obesity when compared with the lowest initial score trajectory. The southern dietary pattern and the modern dietary pattern trajectories of participants in Group 2 (OR = 0.64; 95% CI: 0.51, 0.81; OR = 0.76; 95% CI: 0.63, 0.91) and Group 3 (OR = 0.71; 95% CI: 0.54, 0.91; OR = 0.64; 95% CI: 0.44, 0.90) were associated with lower risk of overweight/obesity when compared with Group 1. We observed that dietary pattern trajectories have different associations with overweight/obesity among Chinese adults.

## 1. Introduction

With economic and social development in China comes a growing burden of overweight and obesity [1]. More than half of adults are overweight or obese, and the rate continues to rise rapidly among all age groups, according to the 2020 Report on the Nutrition and Chronic Disease Status of Chinese Residents released by China’s National Health Commission. The fundamental cause of overweight and obesity is an energy imbalance driven by a multitude of physiological, environmental, and behavioral factors [2,3,4]. Among them, diet plays an important role [5,6].

Dietary pattern analysis is considered a complementary approach to provide more practical and meaningful diet information than individual food items, which is why it has been increasingly applied for investigating diet and its relationship to health outcomes [7,8]. Several studies have assessed the association of overweight/obesity with dietary patterns among Chinese adults [9,10,11,12,13,14]. However, the previous studies were based either on cross-sectional surveys or on provincial data. Additionally, there is still scarce literature with regard to the change in dietary patterns over time and the obesity risks [15].

China is in a nutrition transition, with a Westernization of dietary patterns over recent decades [16]. It is essential to understand the health outcomes of different dietary pattern trajectories over time. The group-based trajectory model approach, which has been used in the field of nutritional epidemiology, identifies groups of individuals following similar trajectories of a single variable within a study population [17]. The trajectory group could provide a comprehensive representation of the long-term exposure. To our knowledge, studies that captured longitudinal changes in dietary patterns are considerably less frequent, and association has not been well investigated in large populations. Therefore, the objectives of this study were to explore the long-term trajectories of dietary patterns and examine the prospective association between different trajectory groups and the risk of overweight/obesity among Chinese adults.

## 2. Materials and Methods

### 2.1. Study Design and Subjects

We used data collected in the China Health and Nutrition Survey (CHNS), which was an ongoing longitudinal study initiated in 1989 and followed up in 1991, 1993, 1997, 2000, 2004, 2006, 2009, 2011, 2015, and 2018. The CHNS used a multistage random cluster process to draw the sample in 15 provinces that varied in demography, geography, economic development, and public resources [18]. We included adults aged 18 years and above at any wave with at least three waves with complete dietary data and body mass index (BMI). We excluded pregnant or lactating women, those having implausible energy intakes (<800 kilocalories (kcal) per day or >6000 kcal for men and <600 kcal or >4000 kcal for women), and those having unrealistic demographic, socioeconomic, anthropometric, and dietary data. The total number of participants and observations were 9299 and 50,374, respectively. The Institutional Review committees of the University of North Carolina at Chapel Hill and the National Institute for Nutrition and Health, Chinese Center for Disease Control and Prevention, approved the survey (No. 201524). Participants provided their written, informed consent.

### 2.2. Measurement of Variables

All waves of the CHNS have obtained clinical, dietary, anthropometric, and all other individual data from each household member. We assessed dietary intake at the individual level by using three consecutive 24-h dietary recalls (two weekdays and one weekend day) in each wave of CHNS. The participants were asked to list the types and amounts of food and beverage items they consumed during a 24-h period. Trained interviewers used standard forms to record the dietary recalls in household interviews. We used a questionnaire to collect several demographic and lifestyle covariates, such as age, gender, and living area, as well as time-varying covariates such as education level, smoking status, alcohol intake, physical activity, and individual income. BMI was calculated using height and weight measurements. In the present study, overweight/obesity was defined as BMI ≥ 24 kg/m^2^ [19].

### 2.3. Statistical Analysis

We conducted factor analysis to derive food patterns according to 19 foods or food groups (Supplementary Materials, Table S1). We used the average intake (g/day) as the input value in the analysis. More details on the methods can be found elsewhere [16].

Group-based trajectory modeling was used to identify dietary pattern trajectories between 1991 and 2018. The number of groups was determined based on some criteria, including their interpretability and better model fit with lower Bayesian information criterion (BIC) [20]. We constructed three-level mixed-effect logistic regression models to assess the risk of overweight/obesity in relation to trajectories of dietary patterns. We calculated odds ratios (ORs; 95% CIs) and we constructed two sequential models. Model 1 adjusted for baseline age, gender, living areas, individual income, education level, physical activity, smoking status, alcohol intake, and energy intake. Model 2 further adjusted for baseline BMI.

We performed all analyses using SAS (Version 9.4, SAS Institute Inc., Cary, NC, USA) and Stata/SE (STATA, Version 15.0, StataCorp, College Station, TX, USA), and we defined statistical significance as *p* < 0.05.

## 3. Results

### 3.1. Dietary Patterns

We derived three dietary patterns using factor analysis (Supplementary Materials, Table S2). Factor 1 was characterized by the food items rice, vegetables, and pork and was named the southern pattern. Factor 2, characterized by high intakes of fruits, dairy products, and processed foods, was called the modern pattern. Factor 3, characterized by high intakes of organ meats, poultry, pork and other livestock meat, was thus called the meat pattern.

### 3.2. Trajectories of Dietary Patterns

**Figure 1:**
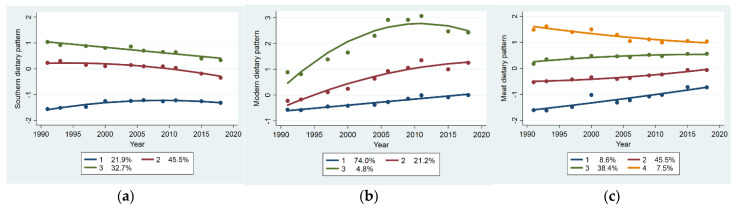
Dietary pattern score trajectory groups from 1991 to 2018: (**a**) southern dietary pattern score trajectory groups; (**b**) modern dietary pattern score trajectory groups; (**c**) meat dietary pattern score trajectory groups.

Trajectories of dietary patterns from 1991 to 2018 are shown in Figure 1. We identified three trajectory groups of the southern dietary pattern. Group 1 (21.9%) had a slight increase and remained below-average score. Group 2 (45.5%) had an initial above-average score and a moderate decrease thereafter. Group 3 (32.7%) had a high initial score and a rapid decrease thereafter. Three trajectory groups of the modern dietary pattern were identified. Group 1 (74.0%) and Group 2 (21.2%) had similar initial below-average scores. Group 1 showed a slight increase and remained below average. Group 2 showed a moderate increase and remained above average. Group 3 (4.8%) had an initial above-average score and rapid increase, with a slight decrease thereafter. We identified four trajectory groups of the meat dietary pattern. Group 1 (8.6%) had the lowest initial score and a rapid increase thereafter. Group 2 (45.5%) had an initial below-average score and a slight increase thereafter. Group 3 (38.4%) had an initial above-average score and a slight increase thereafter. Group 4 (7.5%) had the highest initial score and a slight decrease thereafter.

### 3.3. The Characteristics of the Participants at Baseline

The characteristics of the participants in the analysis by dietary pattern trajectories are given in Table 1. We observed significant differences in age, gender, education level, individual income, living areas, smoking status, physical activity, energy intake, and BMI among trajectory groups of the dietary patterns. For the southern dietary pattern, participants in Group 3 had the youngest age, the highest energy intake, and the lowest BMI. Group 3 had the lowest proportion of females, high education level, and urban residents and the highest proportion of medium income, current smokers, current alcohol drinkers, and high physical activity. For the modern dietary pattern, participants in Group 3 had the oldest age, the lowest energy intake, and the highest BMI. Group 3 had the lowest proportion of current smokers and high physical activity and the highest proportion of females, high education level, urban residents, and high income. For the meat dietary pattern, participants in Group 4 tended to be the youngest. Group 4 had the lowest proportion of females and high physical activity and the highest proportion of high education level, urban residents, high income, current smokers, and current alcohol drinkers.

### 3.4. Trajectories of Dietary Patterns and Overweight/Obesity

As Table 2 shows, there was positive association between risk of overweight/obesity and the meat dietary pattern. Comparing Group 4 with Group 1, ORs (95% CI) of overweight/obesity were 1.63 (1.04, 2.54) when adjusted for all potential confounders. We found that the southern dietary pattern trajectories of participants in Group 2 and Group 3 were associated with lower risk of overweight/obesity when compared with Group 1. The ORs (95% CI) were 0.64 (0.51, 0.81) for Group 2 and 0.71 (0.54, 0.91) for Group 3. We also found that the modern dietary pattern trajectories of participants in Group 2 and Group 3 were associated with lower risk of overweight/obesity when compared with Group 1. The ORs (95% CI) were 0.76 (0.63, 0.91) for Group 2 and 0.64 (0.44, 0.90) for Group 3.

## 4. Discussion

In this study, we identified different trajectories for three dietary patterns among Chinese adults during the 28-year period from 1991 to 2018. Participants who followed the highest initial score and a slight decrease trajectory of the meat dietary pattern were associated with higher risk of overweight/obesity when compared to those with the lowest scores. Moreover, we observed an inverse association between trajectories of the southern and modern dietary patterns with risk of overweight/obesity. To the best of our knowledge, the present study is the first that investigates the long-term longitudinal association of dietary pattern trajectories and overweight/obesity in a large-scale Chinese population.

China is facing a Westernization of dietary patterns in a nutrition transition [16]. Individuals may have a similar change in dietary pattern over time, but they could have begun at a different level and changed at a different pace. It is important to understand if the associated health outcomes for these dietary pattern trajectories are different. In this study, we used a group-based trajectory model to characterize the individual trajectories in dietary pattern scores. Few studies have investigated the change in dietary pattern trajectories [21,22,23]. It is difficult to compare the trajectory of the dietary patterns because of differing food cultures and study methods. However, what we identified here showed some similarities with a previous study among Chinese adults [15].

Red and processed meats are the foods that most frequently contributed to unhealthy dietary patterns [24]. Unlike in many other countries, fresh red meat was the main component of total meats in China, where fatty fresh pork accounted for the majority of red meat intake [25]. Moderate amounts of meat represent an important part of a healthy balanced diet, supplying high-quality protein and many micronutrients, but larger amounts can have adverse health effects [26]. The existing literature has shown positive associations between meat intake and risk for obesity [27,28]. The present study found that participants who followed the highest initial score and a slight decrease trajectory of the meat dietary pattern were positively associated with overweight/obesity when compared with the lowest initial score trajectory. Higher score means higher adherence to the dietary pattern. The results suggest that long-term higher adherence to the meat dietary pattern may increase the risk of overweight/obesity.

Our study showed that the higher score trajectory of the modern dietary pattern was associated with lower risk of overweight/obesity. The modern pattern in the present study had high loadings mostly for snacks, including fruits, dairy products, and processed foods. The protective role of the modern pattern may be owing to the benefits of fruits, dairy products, and nuts. It has previously been proposed that increasing the daily consumption of fruit is correlated to weight loss [29]. A meta-analysis indicates that consumption of dairy products may be associated with a decreased risk of obesity [30]. Studies also show that incorporating nuts into diets does not lead to weight gain and may aid weight maintenance [31,32]. In contrast, epidemiological evidence has demonstrated that the presence of processed foods, which are typically high in salt, fat, and sugar, lead to a high prevalence of overweight/obesity [33]. It is plausible that the healthy components of food items in the dietary pattern may counter the detrimental effects of processed foods.

The southern pattern represents a traditional dietary habit in southern China, where people are more likely to eat rice as a staple food with dishes [16]. Other studies also have reported the association between similar dietary patterns with overweight/obesity in the Chinese population [9,10,14]. Our findings add to accumulating evidence that the traditional southern pattern is related to a lower risk of overweight/obesity. One possible mechanism is that the consumption of this diet appears to be a marker of diet diversity. Another explanation is that rice and vegetables are considered to have a protective effect against obesity [34,35].

There are many strengths in our study. CHNS provided a unique opportunity to explore the longitudinal dietary pattern–disease relationship because of its large participant size and longer follow-up. A comprehensive range of potential confounders with wide temporal and spatial variation enabled us to better understand the impact of changing dietary patterns on obesity. Most importantly, we adopted a group-based trajectory model to classify the dietary patterns into groups sharing common changing dietary characteristics, which could distinguish the health outcomes of different trajectories for a dietary pattern. There are also several limitations. First, the statistical methods we used to define the dietary patterns are somewhat subjective. Second, the 24-h dietary recall method cannot generally evaluate usual dietary intake.

## 5. Conclusions

In conclusion, we observed a positive association between the meat pattern and overweight/obesity among Chinese adults. We also found that the southern and modern patterns were inversely associated with risk of overweight/obesity. Given that people are experiencing a transition of diet, these findings have important implications for preventing overweight/obesity. From a public health perspective, there is an urgent need for interventions to draw attention to unhealthy dietary patterns and promote the adoption of healthy dietary patterns.

## Figures and Tables

**Table 1:** Characteristics of participants by dietary pattern trajectories at baseline.

	Southern Dietary Pattern				Modern Dietary Pattern				Meat Dietary Pattern				
	Group 1	Group 2	Group 3	*p*	Group 1	Group 2	Group 3	*p*	Group 1	Group 2	Group 3	Group 4	*p*
N	2021	4382	2896		7045	1818	436		662	4396	3728	513	
Age (years)	40.6 ± 14.5	41.8 ± 14.8	38.0 ± 12.9	<0.0001	40.0 ± 14.3	40.3 ± 13.9	46.3 ± 14.9	<0.0001	39.6 ± 12.9	40.9 ± 14.2	40.3 ± 14.7	37.5 ± 13.1	<0.0001
Female, %	51.0	57.7	39.0	<0.0001	49.7	52.1	54.4	0.0487	41.5	52.0	51.8	38.0	<0.0001
Education, high %	18.7	26.2	17.0	<0.0001	14.5	40.1	59.8	<0.0001	6.8	15.4	30.3	33.5	<0.0001
Income, %				<0.0001				<0.0001					<0.0001
Low	41.7	24.1	35.1		37.5	13.7	5.5		49.0	38.4	22.5	12.9	
Medium	29.6	31.3	34.1		34.8	24.7	12.9		34.0	33.4	29.7	30.7	
High	28.7	44.6	30.8		27.7	61.6	81.6		17.0	28.2	47.8	56.4	
Urban, %	25.7	38.8	16.8	<0.0001	22.4	46.5	63.5	<0.0001	2.7	20.8	41.1	46.8	<0.0001
Current smoker, %	33.6	29.0	38.3	<0.0001	34.3	29.6	24.5	<0.0001	42.8	32.5	30.2	43.9	<0.0001
Current drinker, %	36.9	33.9	42.4	<0.0001	37.3	36.8	36.7	0.8913	38.7	36.6	36.0	48.6	<0.0001
Physical activity, %				<0.0001				<0.0001					<0.0001
Low	31.8	41.6	25.1		28.4	50.7	61.5		13.2	27.4	43.5	55.3	
Medium	36.1	31.3	37.0		34.8	32.2	31.3		33.1	35.0	33.4	33.0	
High	32.1	27.1	37.9		36.8	17.1	7.2		53.7	37.6	23.1	11.7	
Energy (kcal/day)	2377.8 ± 748.4	2206.7 ± 692.4	2514.4 ± 699.0	<0.0001	2362.5 ± 725.3	2284.0 ± 705.8	2204.3 ± 656.0	<0.0001	2594.4 ± 746.5	2399.0 ± 728.3	2221.6 ± 686.2	2362.0 ± 699.5	<0.0001
BMI (kg/m^2^)	21.2 ± 1.7	20.9 ± 1.8	20.7 ± 1.7	<0.0001	20.8 ± 1.7	21.2 ± 1.7	21.3 ± 1.8	<0.0001	21.0 ± 1.6	20.9 ± 1.7	20.8 ± 1.8	20.8 ± 1.7	0.0072

Values are mean ± SD for continuous variables and percentage for categorical variables. ANOVA for continuous variables and χ^2^ for categorical variables.

**Table 2:** ORs (95% CI) of overweight/obesity across dietary pattern trajectory groups.

	Group 1	Group 2	Group 3	Group 4
Southern dietary pattern				
Model 1	1.00 (ref)	0.45 (0.35, 0.56) ***	0.51 (0.38, 0.67) ***	/
Model 2	1.00 (ref)	0.64 (0.51, 0.81) ***	0.71 (0.54, 0.91) **	/
Modern dietary pattern				
Model 1	1.00 (ref)	0.83 (0.68, 1.00)	0.65 (0.45, 0.93) *	/
Model 2	1.00 (ref)	0.76 (0.63, 0.91) **	0.64 (0.44, 0.90) *	/
Meat dietary pattern				
Model 1	1.00 (ref)	1.37 (1.05, 1.76) *	1.37 (1.03, 1.81) *	2.20 (1.47, 3.26) ***
Model 2	1.00 (ref)	1.26 (0.88, 1.78)	1.26 (0.85, 1.84)	1.63 (1.04, 2.54) *

* *p* < 0.050, ** *p* < 0.010, *** *p* < 0.001. All models were constructed using three-level mixed-effects logistic regression. Model 1 adjusted for baseline age, gender, living area, individual income, education level, physical activity, smoking status, alcohol intake, and energy intake. Model 2 additionally adjusted for baseline BMI.

## Data Availability

Data sharing is not applicable to this article.

## Supplementary Materials

The following are available online at https://www.mdpi.com/article/10.3390/nu13082835/s1. Table S1: Food groups in the factor analysis. Table S2: Factor loadings for dietary patterns.
