# Supplementary material for: Trajectories of Dietary Patterns and Their Associations with Overweight/Obesity among Chinese Adults: China Health and Nutrition Survey 1991–2018
Source: Nutrients. 2021 Aug 18;13(8):2835. doi: 10.3390/nu13082835 (PMC8401187; doi:10.3390/nu13082835)
Supplement: Supplementary file 1 [file nutrients-13-02835-s001.zip › nutrients-1287397-SI.pdf]

Table S1. Food groups in the factor analysis

Food or food groups	Foods included in the group
Rice	Round grained rice, long grained rice, and products
Wheat	Wheat flour and products
Other cereals	Corn, barley, millet, and products
Starchy roots and tubers	Potato, sweet potato, starch, and products
Legumes	Soybean, and products
Fungi and algae	Mushroom, kelp, laver
Vegetables	Cabbage, eggplant, carrot, pepper, lettuce, other vegetables
Fruits	Apple, pear, peach, date, grape, watermelon, orange, other fruit
Pork	Pork and pork products
Other livestock meat	Beef, game, lamb, and meat products
Poultry	Chicken, duck, goose
Organ meats	Organ meats
Fish and seafood	Fish, shrimp, crab, shellfish
Dairy products	Milk, yogurt and products
Eggs	Eggs and products
Nuts and seeds	Walnut, almond kernel, peanut, and others
Cakes, cookies and pastries	Cakes, cookies and pastries, bread, biscuit
Fast foods	Convenience food, hamburger, pizza, sandwich, French fries
Ethnic foods	Cold noodles, pancakes, and others

Table S2. Factor loadings for dietary patterns identified by factor analysis

	Factor1	Factor2	Factor3
Rice	0.81		
Vegetables	0.52		
Fish and seafood	0.29	0.28	0.25
Other cereals	-0.45		-0.25
Wheat	-0.69		-0.26
Fruits		0.61	
Dairy products		0.60	
Cakes, cookies and		0.51	
Eggs		0.46	
Nuts and seeds		0.35	
Fungi and algae		0.31	
Fast foods	-0.25	0.31	
Organ meats			0.47
Poultry			0.43
Pork	0.35		0.40
Other livestock meat			0.39
Starchy roots and			-0.57
Legumes	/	/	/
Ethnic foods	/	/	/
Variance explained (%)	11.4	9.8	6.1

Absolute values < 0.25 are not presented
